# Arbuscular mycorrhizal fungi improve the disease resistance of *Lycium barbarum* to root rot by activating phenylpropane metabolism

**DOI:** 10.3389/fpls.2024.1459651

**Published:** 2024-09-04

**Authors:** Nan Li, Wei Chen, Bin Wang, Chongqing Zhang, Yupeng Wang, Ruiyun Li, Yuke Yan, Jing He

**Affiliations:** ^1^ College of Forestry, Gansu Agricultural University, Lanzhou, China; ^2^ Wolfberry Harmless Cultivation Engineering Research Center of Gansu Province, Lanzhou, China

**Keywords:** root rot of *Lycium barbarum*, *Rhizophagus intraradices*, phenylpropane metabolism, pathogenesis-related protein, disease resistance

## Abstract

Root rot is one of the common diseases of *Lycium barbarum*. Pathogens can cause devastating disasters to plants after infecting host plants. This study investigated the effect of arbuscular mycorrhizal fungi (AMF) *Rhizophagus intraradices* inoculation on phenylpropane metabolism in *L. barbarum* and evaluated its resistance to root rot. The experiment was set up with AMF inoculation treatments (inoculated or not) and root rot pathogen-*Fusarium solani* inoculation treatments (inoculated or not). The results showed that AMF was able to form a symbiosis with the root system of *L. barbarum*, thereby promoting plant growth significantly and increasing plants’ resistance to disease stress. The plant height of AMF-colonized *L. barbarum* increased by 24.83% compared to non-inoculated diseased plants. After inoculation with AMF, the plant defense response induced by pathogen infection was stronger. When the enzyme activity of the leaves reached the maximum after the onset of mycorrhizal *L. barbarum*, phenylalanine ammonia-lyase, cinnamic acid-4-hydroxylase, and 4-coumaric acid-CoA ligase increased by 3.67%, 31.47%, and 13.61%, respectively, compared with the non-inoculated diseased plants. The products related to the lignin pathway and flavonoid pathway downstream of phenylpropane metabolism such as lignin and flavonoids were also significantly increased by 141.65% and 44.61% compared to nonmycorrhizal diseased plants. The activities of chitinase and β-1,3-glucanase increased by 36.00% and 57.96%, respectively. The contents of salicylic acid and jasmonic acid were also 17.7% and 31.63% higher than those of nonmycorrhizal plants in the early stage of plant growth, respectively. The results indicated that AMF significantly promoted plant growth and enhanced disease resistance by increasing enzyme activities and the production of lignin and flavonoids.

## 1 Introduction

*Lycium barbarum* L. is a deciduous shrub belonging to the genus *Lycium* of the Solanaceae family. It is mainly distributed in the arid and semi-arid environments in northwest and north China, as well as Eurasia, Africa, and North and South America (Gong et al., 2022). It has a wide range of cultivation scales in China, mainly concentrated in Nei Monggol, Gansu, Ningxia, Shaanxi, and Qinghai provinces (Zhang et al., 2018). As an excellent shrub for soil and water conservation, *L. barbarum* has significant ecological value, including drought resistance, windbreak and sand fixation capabilities, and soil improvement properties. Its roots, stems and leaves are used as medicine, and its fruits are rich in bioactive substances (polysaccharides, minerals, carotenoids, and polyphenols), making them excellent functional food and natural medicine (Gao et al., 2017; Wang et al., 2018; Yu et al., 2023).

With the continuous expansion of the cultivation scale of *L. barbarum*, the occurrence of *L. barbarum* diseases is becoming more and more serious. Root rot is one of the common diseases in *L. barbarum*, mainly caused by *Fusarium solani*. Pathogenic fungi can bring devastating disasters to plants after infecting host plants, which not only seriously affects the quality of *L. barbarum*, but also poses a huge threat to local economic development. In the early stages of the disease, the roots swell, and the swelling intensifies in the middle stage with a small amount of defoliation. In the later stage, the roots completely rot and show reddish brown, and the plants shed a large amount of leaves until they wither and die (He et al., 2023; Zhang et al., 2023a). In the main cultivation areas of *L. barbarum*, the incidence of root rot can reach 50%, resulting in serious yield and income reduction, drastically hindering the development of the *L. barbarum* industry (Zhu et al., 2023). So far, chemical control remains the most important method for managing this disease. However, long-term use of chemical agents can lead to increased resistance in pathogenic fungi and environmental pollution. Biological control has attracted more and more attention because of its safety, environmental protection, no residue, and other advantages that chemical control can’t surpass. At present, the use of biocontrol microorganisms instead of synthetic fungicides has become a hot topic for many scholars, progressive farmers, cultivators, and even end users.

Arbuscular Mycorrhizal Fungi (AMF) are a group of widely distributed soil fungi that typically form symbiotic relationships with about 85% of terrestrial plants (Khaliq et al., 2022). In forestry production and ecological restoration and protection, AMF, as a ubiquitous symbiotic microorganism with plants, has a high application value and potential production and development prospects (Qu et al., 2022b). Studies have shown that AMF can respond to biotic stresses such as diseases (Devi et al., 2022) and pests (Wang et al., 2023b), and abiotic stresses such as drought (Zou et al., 2021), waterlogging (Xu et al., 2024), low temperature (Li et al., 2020) and salinity (Li et al., 2024) by changing root morphological structure, competing with pathogens for photosynthetic products and living space, activating disease-related defense enzyme systems, and regulating the formation of secondary metabolites of host plants. Aseel et al. (2019) found that after inoculation with AMF, the gene expression level of the phenylpropane synthesis pathway in diseased tomatoes was up-regulated, which reduced the severity of tomato mosaic disease. Pre-inoculation with *Glomus versiformme* significantly alleviated *Fusarium* wilt caused by *Fusarium oxysporum* (Pu et al., 2022). Wang et al. (2022) studied the differentially expressed genes of apple plants inoculated with AMF at the transcriptional level after infection with *F. solani* compared with non-inoculated plants, and found that *MdWRKY40* played an important role in the resistance of mycorrhizal apple seedlings to pathogen infection. At the same time, it was also found that inoculation of AMF significantly increased the resistance of apples to *Neonectria ditissima* (Despina et al., 2018). Therefore, in the context of sustainability of ecosystem health, the application of AMF is expected to be a promising biocontrol method due to their ability to promote plant growth and enhance disease resistance (George and Ray, 2023).

The phenylpropane pathway (PPP) is one of the important defense pathways in plants (Despina et al., 2018). A large number of studies have shown that the activity of key enzymes in the PPP, the content of phenylpropanoid compounds, and their derivatives are closely related to the strength of plant disease resistance (Dong and Lin, 2021). At the same time, PPP is also involved in the synthesis of plant disease-resistant hormones. For example, the accumulation of salicylic acid (SA) can activate plant disease-resistant immune signaling pathways and comprehensively regulate plant disease-resistant immune systems (Vlot et al., 2009). Wei et al. (2017) found that hot air treatment induced resistance to *Alternaria alternata* and *Botrytis cinerea* by activating the PPP in cherry tomato fruits. Ge et al. (2018) confirmed that ϵ-poly-L-lysine treatment enhanced the resistance of apple fruits to *Penicillium expansum* by activating reactive oxygen species (ROS) metabolism and PPP. Qu et al. (2022) found that melatonin enhances the postharvest resistance of blueberry fruits to *Valsa* canker by mediating the jasmonic acid (JA) signaling pathway and PPP (Qu et al., 2022a). At present, the research on *L. barbarum* root rot mainly focuses on the pathogenesis (Gao et al., 2022), antagonistic fungi screening and so on (Wang et al., 2023a). Our previous study found that *Rhizophagus intraradices* can form a good symbiotic system with *L. barbarum* and enhance the resistance to root rot (Li et al., 2022). However, how *R. intraradices* influences the PPP of *L. barbarum* and thereby induces it to improve disease resistance has not been reported. Therefore, this study mainly focused on the PPP, growth parameter measurements, PPP pathway-related enzyme activities, pathogenesis-related proteins, related enzyme activities in signal substances, and key substance contents of *L. barbarum*, to reveal the mechanism of AMF-induced *L. barbarum* resistance to root rot from the perspective of PPP, and provide a theoretical basis for the biological control of *L. barbarum* root rot.

## 2 Materials and methods

### 2.1 Materials

The tested AMF agent was *Rhizophagus intraradices* BGC-BG09, which was provided by the Institute of Plant Nutrition and Resources of Beijing Academy of Agriculture and Forestry Sciences. Maize was used as the host for propagation, and the propagated substrate was used as inoculum which contained spores, hyphae, and fine root segments. Subsequently, we isolated and screened the spores using wet sieving and sucrose centrifugation, stained them following the method described by Kumar et al. (2008), and then observed and counted them under a somatic microscope to determine the number of viable spores present in the inoculum.

*F. solani* was isolated from the diseased plants of typical *L. barbarum* root rot, and preserved in the forest protection laboratory of Forestry College of Gansu Agricultural University after a pathogenicity test. Before inoculation, the isolated fungus was activated by sub-culturing on a PDA plate and stored at 4°C. 10 mL of sterile water and two drops of Tween-80 was added to the purified *F. solani* after multiple cultures and gently shook it to ensure the conidia were completely dislodged. The spore count was then performed using a hemocytometer. When using the hemocytometer, the number of spores in each small square was first measured and then converted to the number of spores per milliliter of the fungal suspension. Based on the counting results, the suspension was diluted to 1×10^7^ spores/mL for later use.

### 2.2 Experimental design

The pot experiment was carried out in the economic forest teaching and scientific research practice base of Forestry College of Gansu Agricultural University from March 2023 to September 2023. To avoid the presence of native AMF, sterilized soil was used in all the treatments. The one-year-old seedlings of *L. barbarum* of the same size were selected and transplanted into a 35 cm × 24 cm pot (disinfected with 0.5% sodium hypochlorite) for treatment. In the inoculation group, 10 g of *R. intraradices* inoculum (approximately 100 viable spores per gram) was evenly spread in the pot at the base of root contact before *L. barbarum* transplantation ensuring full contact with the roots. In the non-inoculation group, the same amount of inactivated *R. intraradices* inoculum (autoclaved at 121°C for 2 h) was added following the method used for inoculated pots. and 10 mL microbial filtrate (0.2 μm microporous membrane) of the *R. intraradices* inoculum was added to ensure the consistency of microbial flora. After transplantation, normal watering and fertilizer management were performed. *L. barbarum* seedlings were allowed to grow for 30 d of disease-free stress period to establish a good symbiotic relationship with *R. intraradices*. Later *F. solani* was inoculated after determining the formation of mycorrhiza. The pathogen was inoculated by the root-injury method. 100 mL of *F. solani* spore suspension with a concentration of 10^7^ CFU·mL^-1^ was directly injected into the root, and the same amount of sterile water was added into the control group (CK). The environmental conditions during seedling growth were as follows: temperature range of 20-26°C, relative humidity of 65% -90%, and natural light. Maintain a regular supply of water during seedling growth.

A total of 4 treatments were set up: T1 control: neither *R. intraradices* nor *F. solani* inoculation (-R.i-F.s); T2: inoculated with only *R. intraradices* (+R.i-F.s); T3: inoculated with only *F*. *solani* (-R.i+F.s); T4: double inoculation with *R. intraradices* and *F*. *solani* (+R.i+F.s), and replicated 24 pots per treatment totaling to 96 pots. During the experiment, normal watering and fertilizer management of each plant was maintained. The plant growth indices (including leaf number, plant height, stem diameter, etc.) were observed and recorded at 15 d (leaf expansion period), 30 d (flowering period), 60 d (initial fruit period), 90 d (full fruit period) and 120 d (final fruit period) after *F. solani* inoculation. Leaves were collected at different growth stages to determine physiological and biochemical indexes such as disease resistance-related enzyme activity, JA, and SA.

### 2.3 Determination of mycorrhizal colonization rate

Staining was carried out using the Trypan Blue staining method (Eke et al., 2020). Root samples of different periods were collected and cut into root segments of about 1 cm. The root segments were placed in a centrifuge tube, and 3 mL of 0.5% KOH (immersed root segments) was added and kept in a water bath at 90°C for 15 mins. The root samples were dispersed by gently shaking. Later, the root samples were washed with water 3-5 times until the water flow remained no longer yellow. The cleaned root samples were soaked in 1% HCl for 2 min and removed. To the acidified roots in the centrifuge tube, 0.05% Trypan Blue staining solution (distilled water: glycerol: lactic acid=1:1:3) was added to soak and kept in a water bath at 90°C for 30 min, Later the roots in the tubes were soaked again with 1:1 lactic acid glycerol solution for 12 h (to remove excess stain in roots) and mycorrhizal colonization in roots was observed for mycorrhizal colonization under microscope and estimated according to below equation by.


$$\text{Root colonization rate}=\frac{\text{number of infected root segments}}{\text{number of detected root segments}}\times 100\%$$

### 2.4 Determination of morbidity, disease index, and growth parameters

After the inoculation of *F. solani*, a real-time investigation of *L. barbarum* plants was carried out. After *L. barbarum* shows symptoms of disease, the number of diseased *L. barbarum* leaves was investigated at different growth periods, respectively. The number of diseased leaves was recorded, and the incidence and disease index were calculated according to the corresponding grading standards (Gu et al., 2009).

The plant height and basal diameter of seedlings were measured by tape and vernier caliper, respectively. After the leaves were picked, fresh weight was weighed, dried to constant weight at 65°C in the oven and dry weight was weighed.


$$morbidity=\frac{Number\;of\;diseased\;plants}{Investigate\;the\;number\;of\;plants}\times 100\%$$

$$\begin{array}{c} disease\;index\\ =\sum \frac{Number\;of\;diseased\;plants\;at\;all\;levels\times The\;disease\;grade\;value}{Total\;number\;of\;survey\;plants\times The\;highest\;level\;value} \end{array}\times 100$$

$$\begin{array}{c} control\;effect(\%)\\ =\frac{Control\;disease\;index-treatment\;disease\;index}{Control\;disease\;index} \end{array}\times 100$$

The determination of chlorophyll content referred to the method of Ronen and Galun (1984). 0.2 g fresh leaves were quickly frozen in liquid nitrogen and ground into powder. Dimethyl sulfoxide (analytical purity) was used to extract the powder in a constant temperature water bath at 60°C for 1 h in the dark. The supernatant was collected and the absorbance of chlorophyll a and b was determined at 663 and 645 nm.

### 2.5 Determination of SA and JA

The content of SA and JA was determined using the corresponding enzyme-linked immunosorbent assay (ELISA) kits. The corresponding item numbers are YX-22154P and YX-21810P. The kits were all from Shanghai Youyou Biotechnology Co., Ltd.

### 2.6 Determination of pathogenesis-related protein in *L. barbarum* leaves

The extraction method of enzyme solution was referred to as 2.5.1. Chitinase activity was measured using the corresponding ELISA kit. The corresponding item number of the kit is YX-E22624P, which was obtained from Sino Best Biological Technology Co., Ltd. The extraction of β-1,3-glucanase was determined according to the method of Zhang et al. (2013). The amount of enzyme required for the reaction system to change the absorbance value at 540 nm by 0.01 per hour was an enzyme activity unit (U). The polygalacturonase (PG) was determined according to the method of Cao et al. (2007). The mass of polygalacturonic acid hydrolyzed into galacturonic acid (mg·h^-1^·g^-1^) was expressed as per gram of plant tissue sample (fresh weight) per hour at 37°C. Determination of pectin methylgalacturonase (PMG) was based on the method of Cao et al. (2007), expressed as the mass per gram of plant tissue sample per hour at 37°C catalyzed substrate hydrolysis to produce galacturonic acid (mg·h^-1^·g^-1^).

### 2.7 Determination of enzyme activities and products related to PPP in *L. barbarum* leaves

Phenylalanine ammonia-lyase (PAL) activity was determined according to the method of Khumalo et al. (2017). The amount of enzyme required to change the absorbance value at 290 nm by 0.01 per minute of the reaction system was used as an enzyme activity unit (U). The activity of cinnamic acid-4-hydroxylase (C4H) was determined according to the method of Ackah et al. (2022), the method of enzyme solution extraction was referred to as 2.5.1, and the amount of enzyme required to change the absorbance value at 340 nm by 0.01 per minute of the reaction system was taken as an enzyme activity unit (U). Determination of 4-coumaric acid-CoA ligase (4CL) activity according to the method of Ackah et al. (2022), the amount of enzyme required to change the absorbance value at 333 nm by 0.01 per minute of the reaction system was taken as an enzyme activity unit (U). Determination of chalcone isomerase (CHI) activity according to the method of Latunde-Dada et al. (1987), the amount of enzyme required to change the absorbance value at 290 nm by 0.01 per minute of the reaction system was used as an enzyme activity unit (U). The activities of chalcone synthase (CHS), flavanone hydroxylase (F3H), hydroxycinnamic acid transferase (HCT), cinnamoyl-CoA reductase (CCR), and cinnamyl alcohol dehydrogenase (CAD) were determined using the corresponding ELISA kits. The corresponding item numbers of the kits are YX-E21833P, YX-E22631P, YX-E22626P, YX-E22625P, and YX-E21912P. All of the above kits were derived from Sino Best Biological Technology Co., Ltd. Lignin content was determined by A280 nm·mg protein according to the method of Bonawitz et al. (2014). Contents of flavonoids and total phenols were determined according to the method of Cao et al. (2007). The flavonoid content was expressed as OD_325_ nm/g, and the total phenol content was expressed as the absorbance value of fresh weight per gram plant tissue at 280 nm, and calculated by gallic acid standard curve, expressed as mg g^-1^.

### 2.8 Data processing and analysis

SPSS 26.0 was used to test the homogeneity of variance on the original data. When the Sig value was > 0.05, the variance was considered to be homogeneous, and then one-way ANOVA analysis of variance was performed. Duncan’s method was used for specific *post-hoc* tests, and the significance level was set as α = 0.05. The data were expressed as mean ± standard error (SE). Origin 2021 was used for plotting.

## 3 Results and analysis

### 3.1 *R. intraradices* colonization and infection in *L. barbarum* roots

**Figure 1:**
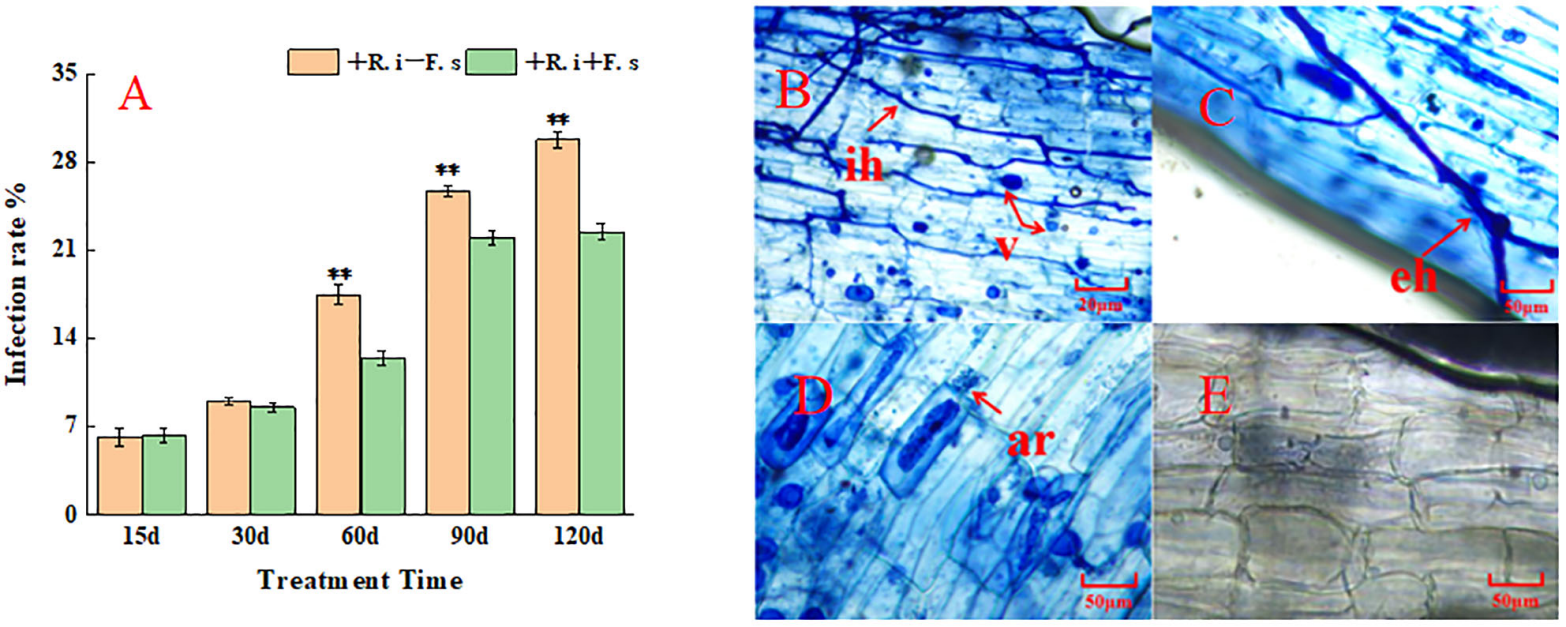
Mycorrhizal colonization rate and infection structure of *L. barbarum* roots at different growth stages. **(A)** Mycorrhizal infection rate; **(B–E)** *R. intraradices* infection structure: ih, intraradical hyphae; eh, exogenous hyphae; v, vesicles; ar, arbuscule.

The colonization rate gradually increased with the change in the plant growth period. The colonization rate of mycorrhizal diseased plants in the middle and late stages was lower than that of plants inoculated with only *R. intraradices*, indicating that the presence of *F. solani* had a negative effect on the colonization of *R. intraradices*(**Figure 1A**). The colonization structure of *R. intraradices*, such as hyphae, arbuscular and vesicles, could be observed after *R. intraradices* inoculation (**Figures 1B–D**). In contrast, no *R. intraradices* colonization was observed in the non-inoculated treatment (**Figure 1E**), the colonization rate of control plants was 0.

### 3.2 Effect of *R. intraradices* on growth parameters, incidence, and disease index of *L. barbarum* under different inoculation treatments

Whether inoculated with *R. intraradices* or not, the *L. barbarum* plants inoculated with *F. solani* would develop disease in the later growth stages. However, the colonization of *R. intraradices* could significantly reduce the incidence and disease index of *L. barbarum*, indicating that the inoculation of *R. intraradices* to the roots of *L. barbarum* plants could significantly enhance the resistance of plants and reduce the occurrence of root rot (**Table 1**).

**Table 1:** Effects of different inoculation treatments on growth parameters of *L. barbarum*.

Days(d)	Treatment	-R. i-F. s	-R. i+F. s	+R. i-F. s	+R. i+F. s
leaf expansion period	stem thickness/mm	7.93 ± 0.57b	7.07 ± 0.46c	11.77 ± 0.81a	12.35 ± 0.57a
	plant height/cm	60.42 ± 14.63b	59.37 ± 17.37b	80.62 ± 11.63a	85.73 ± 14.63a
	number of leaves	121.53 ± 17.53b	119.33 ± 23.64b	169.78 ± 29.83a	179.63 ± 27.47a
	leaf aspect ratio	3.03 ± 0.71b	3.56 ± 1.85a	3.07 ± 0.54b	3.56 ± 0.47b
	leaf area/cm^2^	6.53 ± 1.39bc	5.19 ± 1.49c	8.13 ± 1.74ab	8.04 ± 1.32a
	leaf blade girth/cm	13.19 ± 1.96bc	12.49 ± 1.22c	15.62 ± 1.93ab	16.26 ± 0.68a
	leaf index	0.15 ± 0.01a	0.13 ± 0.01a	0.13 ± 0.06a	0.12 ± 0.01a
	leaf water content	0.82 ± 0.01a	0.78 ± 0.02a	0.82 ± 0.05a	0.83 ± 0.02a
	Morbidity	0	8.33%	0	4.17%
	disease index	0	8.33	0	4.17
	control effect %	/	/	/	50
flowering period	stem thickness/mm	9.1 ± 1.39b	7.07 ± 1.02b	13.17 ± 1.99a	15.15 ± 1.83a
	plant height/cm	74.42 ± 16.67bc	69.67 ± 21.13c	87.83 ± 15.75ab	91.92 ± 14.63a
	number of leaves	183.92 ± 21.1b	167.33 ± 29.77b	219.58 ± 29.83a	222.42 ± 45.93a
	leaf aspect ratio	3.42 ± 0.71b	4.54 ± 1.85a	3.56 ± 0.54b	3.66 ± 0.47b
	leaf area/cm^2^	7.14 ± 1.59bc	6.1 ± 1.48c	8.23 ± 2.1ab	8.96 ± 1.41a
	leaf blade girth/cm	13.97 ± 1.52bc	13.12 ± 1.36c	15.07 ± 1.81ab	16.14 ± 0.91a
	leaf index	0.14 ± 0.01a	0.14 ± 0.01a	0.15 ± 0.06a	0.14 ± 0.01a
	leaf water content	0.81 ± 0.01a	0.81 ± 0.02a	0.81 ± 0.05a	0.82 ± 0.02a
	Morbidity	4.17%	16.67%	0	8.33%
	disease index	4.17	10.42	0	8.33
	control effect %	/	/	/	20
initial fruit period	stem thickness/mm	9.19 ± 0.81c	7.25 ± 0.82d	14.57 ± 1.01a	12.67 ± 1.58b
	plant height/cm	105.7 ± 17.74ab	93.8 ± 24.01b	118.2 ± 27.96a	104.4 ± 26.13ab
	number of leaves	147.1 ± 34.08b	104.3 ± 30.77c	191.3 ± 23.00a	176.8 ± 40.93ab
	leaf aspect ratio	3.65 ± 0.51a	4.06 ± 0.74a	3.68 ± 0.44a	3.71 ± 0.31a
	leaf area/cm^2^	8.25 ± 292.10b	7.12 ± 238.91b	9.06 ± 203.81ab	10.85 ± 215.98a
	leaf blade girth/cm	14.92 ± 26.37b	14.44 ± 19.96b	15.78 ± 15.36b	17.48 ± 18.4a
	leaf index	0.15 ± 0.01a	0.13 ± 0.02c	0.14 ± 0.01ab	0.14 ± 0.01ab
	leaf water content	0.82 ± 0.01a	0.81 ± 0.05a	0.82 ± 0.04a	0.83 ± 0.04a
	Morbidity	8.00%	29.17%	0	16.67%
	disease index	8.33	20.83	0	16.67
	control effect %	/	/	/	20
full fruit period	stem thickness/mm	11.1 ± 1.72ab	9.93 ± 0.56b	12.59 ± 1.23a	12.52 ± 2.26a
	plant height/cm	107.00 ± 19.52ab	96.00 ± 21.89b	131.33 ± 20.47a	102.17 ± 27.71b
	number of leaves	169.33 ± 18.79a	107.67 ± 37.58b	187.17 ± 29.92a	169.00 ± 15.31a
	leaf aspect ratio	3.93 ± 0.45a	4.02 ± 0.97a	3.72 ± 0.53a	3.84 ± 0.71a
	leaf area/cm^2^	7.48 ± 0.81b	6.09 ± 1.28b	10.25 ± 0.75a	9.54 ± 2.97a
	leaf blade girth/cm	14.9 ± 1.07bc	13.65 ± 1.35c	16.96 ± 0.75ab	16.44 ± 2.14a
	leaf index	0.14 ± 0.01a	0.13 ± 0.02a	0.14 ± 0.01a	0.14 ± 0.01a
	leaf water content	0.77 ± 0.01b	0.8 ± 0.01ab	0.81 ± 0.03ab	0.85 ± 0.1a
	Morbidity	8.33%	45.83%	4.17%	20.83%
	disease index	8.33	23.61	4.17	12.5
	control effect %	/	/	/	47
final fruit stage	stem thickness/mm	10.75 ± 1.21ab	9.75 ± 0.43b	12.73 ± 1.41a	12.48 ± 2.25a
	plant height/cm	113.00 ± 17.74a	102.00 ± 21.89b	127.33 ± 20.26a	110.17 ± 24.41a
	number of leaves	153.31 ± 18.79a	89.37 ± 37.58b	169.27 ± 17.94a	154.00 ± 14.91a
	leaf aspect ratio	4.24 ± 0.14a	4.3 ± 0.26a	3.74 ± 0.57ab	3.46 ± 0.08b
	leaf area/cm^2^	5.24 ± 1.17bc	3.98 ± 0.61c	7.76 ± 1.58a	7.12 ± 0.5ab
	leaf blade girth/cm	12.55 ± 1.25bc	11.07 ± 0.71c	14.32 ± 0.77a	13.8 ± 0.47ab
	leaf index	0.13 ± 0.00ab	0.13 ± 0.01b	0.15 ± 0.02a	0.15 ± 0.00a
	leaf water content	0.79 ± 0.04a	0.72 ± 0.08a	0.72 ± 0.08a	0.77 ± 0.02a
	Morbidity	12.50%	54.17%	4.17%	29.17%
	disease index	12.5	29.17	4.17	16.67
	control effect %	/	/	/	43

The values are mean ± standard error (SE); Different lowercase letters represent significant differences between data in the same column (P<0.05),-R. i-F. s: neither inoculated with R. intraradices nor F. solani; +R. i-F. s: inoculated R. intraradices but not with F. solani; -R. i+F. s: not inoculated with R. intraradices but with F. solani; +R. i+F. s: Double inoculation with R. intraradices and F. solani.

**Figure 2:**
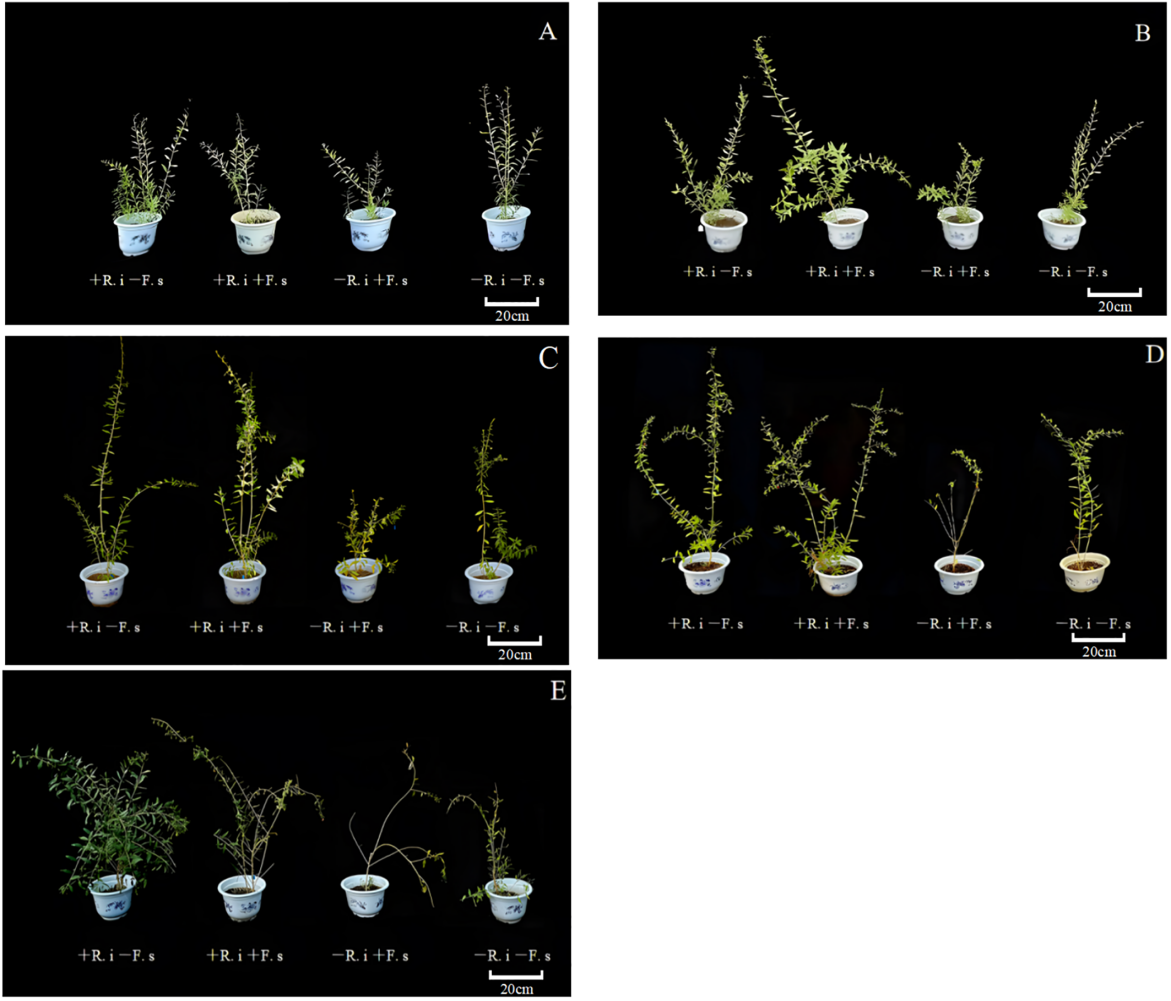
Growth status of *L. barbarum* in different periods. **(A)** 15 d (leaf expansion period); **(B)** 30 d (flowering period); **(C)** 60 d (first fruit stage); **(D)** 90 d (full fruit stage); **(E)** 120 d (final fruit stage).

With the change in the growth potential of *L. barbarum* plants, there was a significant difference between the *L. barbarum* plants colonized by *R. intraradices* and the *L. barbarum* plants in the control group. At the same time, due to the presence of *R. intraradices*, the height of mycorrhizal plants was significantly higher than that of plants only inoculated with *F. solani* at the later stage (**Figure 2**).

### 3.3 Effects of *R. intraradices* on PPP metabolic pathway in *L. barbarum* leaves under different inoculation treatments

#### 3.3.1 Enzyme activities related to the PPP

**Figure 3:**
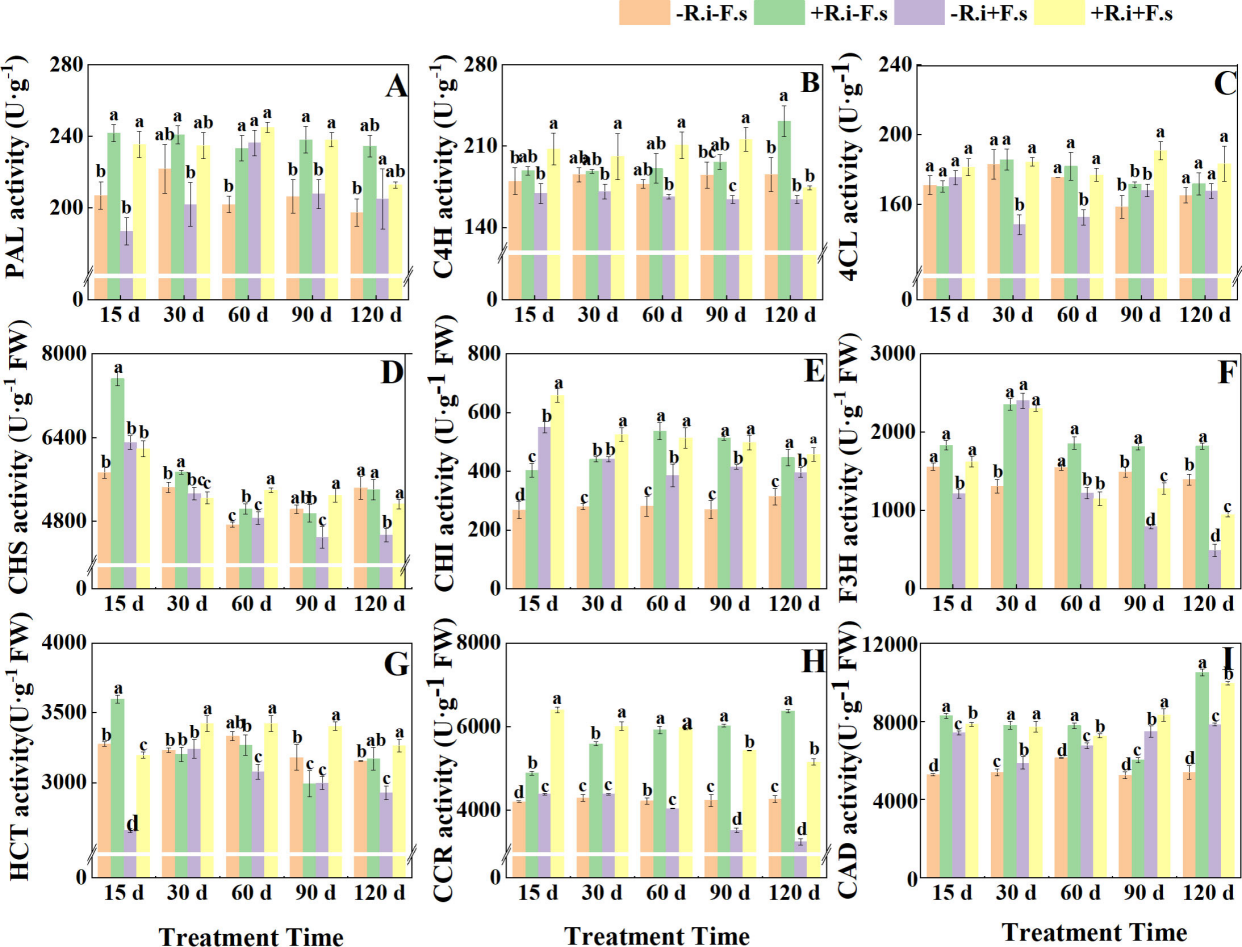
Effects of *R. intraradices* on the activities of PAL **(A)**, C4H **(B)**, 4CL **(C)**, CHS **(D)**, CHI **(E)**, F3H **(F)**, HCT **(G)**, CCR **(H)**, CAD **(I)** in *L. barbarum* leaves under different inoculation treatments. Different lowercase letters indicated significant differences between the control and treatments (p < 0.05).

The two treatments of only inoculation with *R. intraradices* and only inoculation with *F. solani* could induce the increase of PAL activity in leaves (appeared in different periods). After pre-inoculation with *R. intraradices*, PAL activity could be enhanced under *F. solani* stress to alleviate the damage caused by *F. solani*. The treatment of inoculated *F. solani* showed a trend of increasing first and then decreasing, reaching the maximum at 60 days. The enzyme activity of mycorrhizal diseased plants was 3.67% higher than that of non-mycorrhizal diseased plants (**Figure 3A**).

Mycorrhizal *L. barbarum* significantly increased C4H activity by 22.64%, 17.64%, 26.61% and 31.47% under *F. solani* stress. The C4H activity of mycorrhizal plants increased gradually in five periods and reached the maximum at 120 d, which was 41.4% and 33.0% higher than that of non-mycorrhizal diseased plants and mycorrhizal diseased plants, respectively. Because of the inoculation of *F. solani*, the mycorrhizal plants had the opposite trend with the treatment of *R. intraradices* alone in the later stage, which was consistent with the trend of single inoculation of *F. solani* (**Figure 3B**). Under the condition of inoculation with *F. solani*, the 4CL activity of mycorrhizal plants was 3.44%, 24.34%, 15.97% and 13.61% higher than that of non-mycorrhizal plants within 15-90 days (**Figure 3C**).

With the increase in treatment time, the activity of CHS inoculated with *F. solani* showed a decreasing trend. After inoculation with *R. intraradices*, the trend of *F. solani* inoculation treatment was opposite to that of non-*F. solani* inoculation treatment in the later stage, which was consistent with the trend of single *F. solani* inoculation treatment. The enzyme activity of mycorrhizal plants in the mid to late stage (60-120 d) was significantly higher than that of single inoculation of *F. solani* by 11.03%, 17.83% and 12.21%. It can be seen that *R. intraradices* can play a better role in improving CHS activity under disease stress (**Figure 3D**). After inoculation with *F. solani*, the CHI activity of mycorrhizal and non-mycorrhizal seedlings showed a gradual downward trend. Under disease stress, compared with non-inoculated *R. intraradices*, the enzyme activity of AMF-inoculated leaves increased significantly by 19.7%, 19.2%, 33.2%, 20.1% and 15.5% in five periods (**Figure 3E**). The enzyme activity of the treatment only inoculated with *R. intraradices* showed a trend of increasing first and then decreasing, reaching the maximum at 60 d (**Figure 3E**). The F3H activity of the four treatments showed a trend of increasing first and then decreasing as a whole. Under the treatment of *F. solani* inoculation, the difference between *R. intraradices* inoculation and non-AMF inoculation was not significant in the early stages and was significant in the middle and late stages, which was in line with the trend of CHS. *R. intraradices* increased F3H activity as a whole, higher than the other three treatments (**Figure 3F**).

After *R. intraradices* inoculation, HCT activity showed a gradual downward trend regardless of whether the *F. solani* was inoculated or not (**Figure 3G**). Regardless of *R. intraradices* inoculation status, both treatments inoculated with *F. solani* showed a trend of increasing first and then decreasing. Under the stress of disease, the activity of *L. barbarum* inoculated with *R. intraradices* was significantly increased by 20.24%, 5.58%, 11.20%, 13.63% and 11.47% compared with that without *R. intraradices* inoculation (**Figure 3G**). CAD activity was significantly increased by 5.92%, 31.56%, 7.46%, 11.32%, and 26.88% (**Figure 3I**). CCR activity showed a decreasing and then increasing trend in all three treatments except the control (**Figure 3H**). Under disease stress, compared with non-AMF inoculation, the CAD activity of AMF-inoculated plants was significantly increased by 45.76%, 37.00%, 47.67%, 54.20%, and 58.65%.

#### 3.3.2 Products related to the PPP

**Figure 4:**
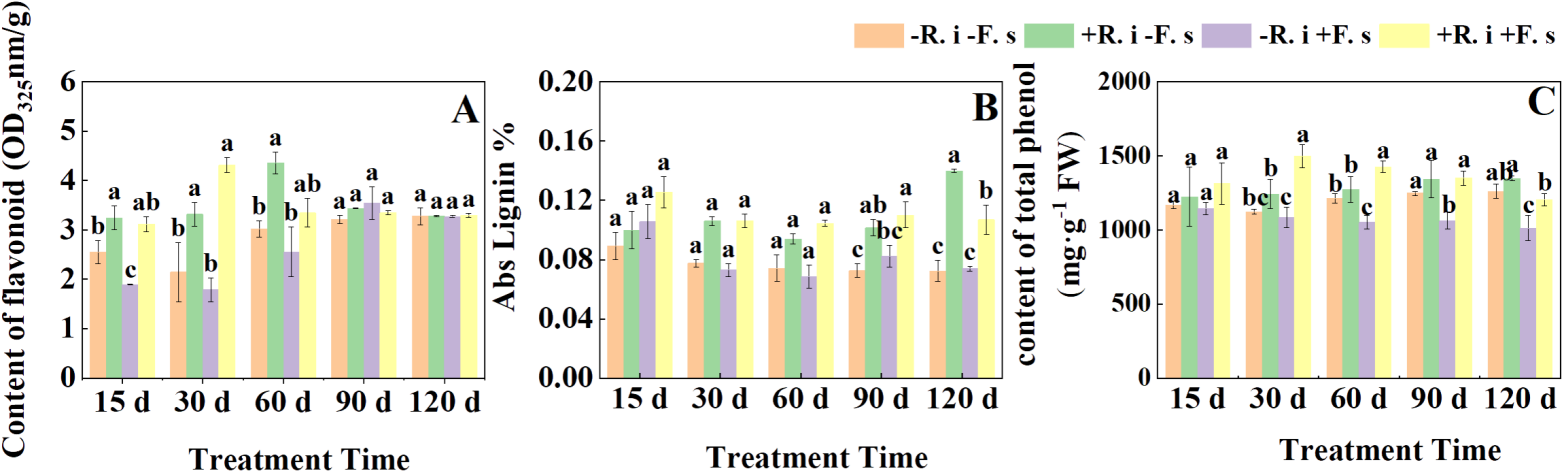
Effects of *R. intraradices* on the content of flavonoid **(A)**, Abs Lignin **(B)**, total phenol **(C)** in *L. barbarum* leaves under different inoculation treatments. Different lowercase letters indicated significant differences between the control and treatments (p < 0.05).

The flavonoid content showed a trend of increasing first and then decreasing as a whole. In the first three periods, compared with non-inoculated *R. intraradices*, the flavonoid content in *L. barbarum* leaves inoculated *R. intraradices* under disease stress was significantly increased by 64.59%, 141.65%, and 30.81% (**Figure 4A**). In contrast, in the first three periods, there was no significant difference in lignin content between mycorrhizal and non-mycorrhizal plants, and increased significantly by 34.13% and 44.61% only in the latter two periods (**Figure 4B**). The total phenol content of the *F. solani* inoculation treatment showed a gradual downward trend, while the content in mycorrhizal plants showed a trend of increasing first and then decreasing due to the infection of *F. solani*. At 15 d, there was no significant difference between the two treatments inoculated with *F. solani*. The total phenol content of mycorrhizal plants was significantly higher than that of non-mycorrhizal plants by 35.79%, 27.01%, and 18.97% (**Figure 4C**).

### 3.4 Effects of *R. intraradices* on the content of plant signaling substances in leaves of *L. barbarum* under different inoculation treatments

**Figure 5:**
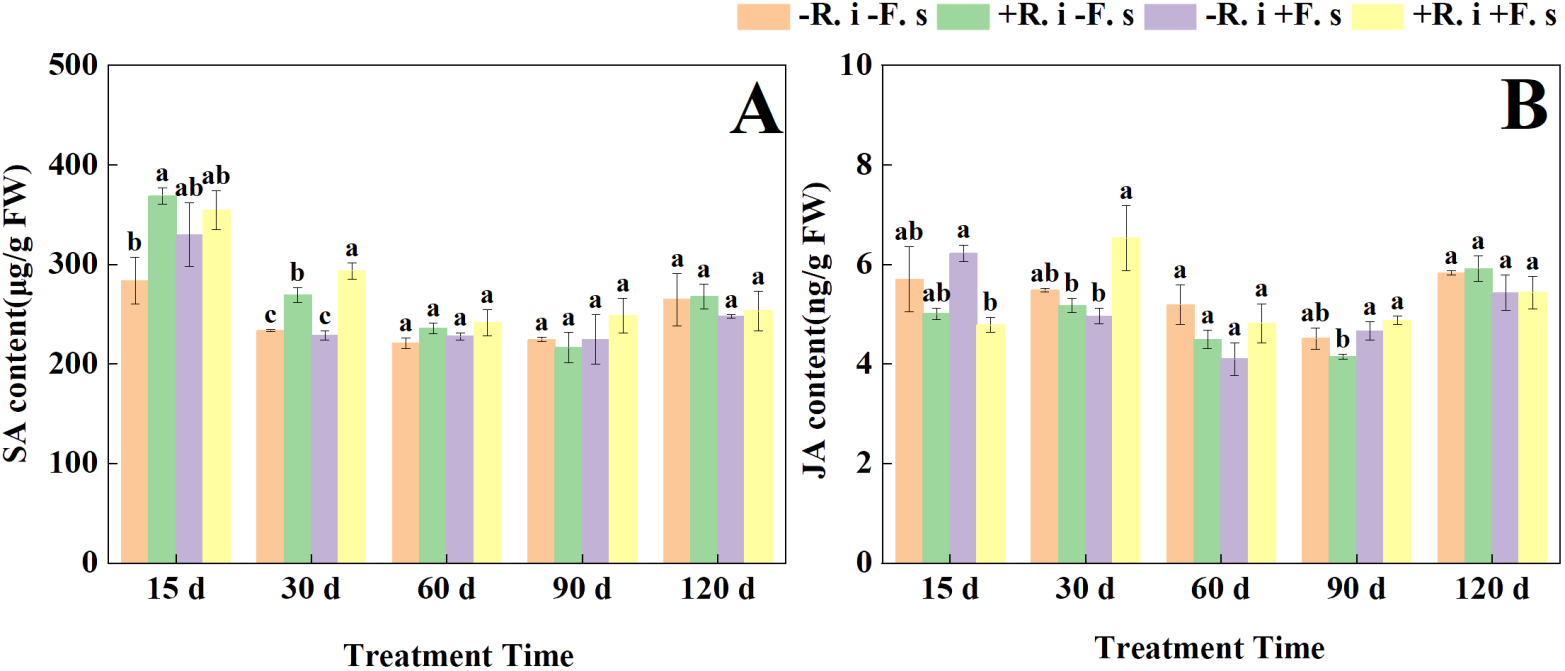
Effects of *R. intraradices* on the content of SA **(A)**, JA **(B)** in *L. barbarum* leaves under different inoculation treatments. Different lowercase letters indicated significant differences between the control and treatments (p < 0.05).

With the prolongation of treatment time, the SA content of each treatment showed a gradual downward trend. The SA content of mycorrhizal plants in the early stage (15 d, 30 d) was 11.7% and 17.7% higher than that of only inoculated *F. solani* treatment (**Figure 5A**). The JA content of all treatments, except for the treatments double inoculated with *R. intraradices* and *F. solani*, showed a general trend of decreasing and then increasing (**Figure 5B**).

### 3.5 Effects of *R. intraradices* on pathogenesis-related proteins of *L. barbarum* leaves under different inoculation treatments

**Figure 6:**
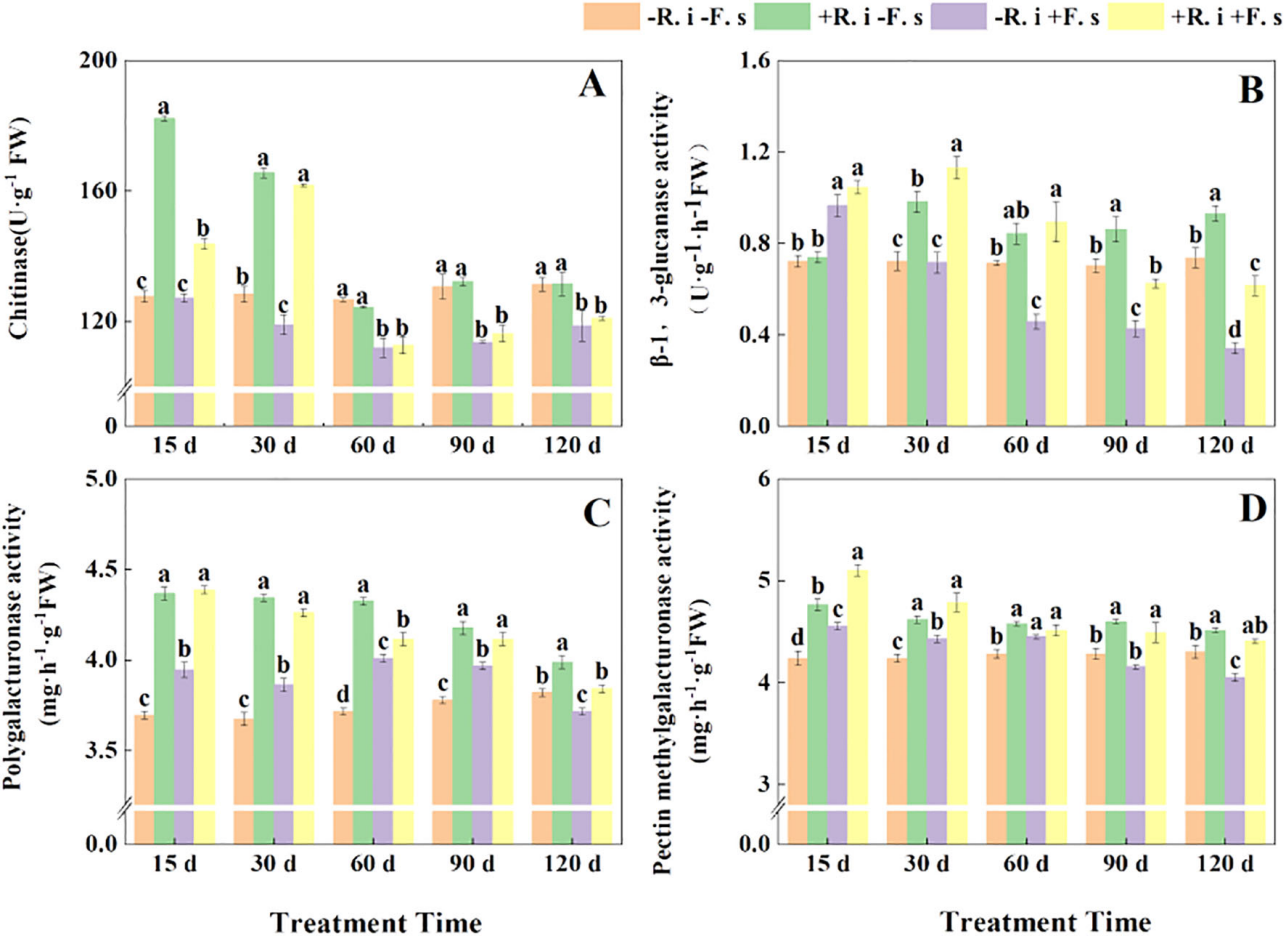
Effects of *R. intraradices* on the activities of chitinase **(A)**, β-1,3-glucanase **(B)**, polygalacturonase **(C)**, pectin methylgalacturonic **(D)** in *L. barbarum* leaves under different inoculation treatments. Different lowercase letters indicated significant differences between the control and treatments (p < 0.05).

In general, chitinase showed a decreasing trend. Under disease stress, compared with non-inoculated treatment, the chitinase activity of mycorrhizal *L. barbarum* increased significantly by 13.0% and 36.0% in the first two periods, and there was no significant difference in the middle and late periods. However, the mycorrhizal *L. barbarum* plants were treated at a high enzyme activity level in five periods, which was higher than that of the mycorrhizal plants treated with pathogenic fungi by 26.74%, 2.31%, 10.39%, 13.67% and 8.73%, respectively (**Figure 6A**).

At the same time, the β-1,3-glucanase activity of the *F. solani* treatment showed a gradual downward trend. After inoculation with *R. intraradices*, the β-1,3-glucanase activity showed a trend of increasing first and then decreasing with or without inoculation with *F. solani*. The enzyme activity of the five periods was higher than that of the treatment inoculated with *F. solani* (**Figure 6B**). The activity of pectin methylgalacturonic acid enzyme in different treatments showed a gradual downward trend. The enzyme activity of mycorrhizal diseased plants in the early stage was higher than that of the other three treatments, while the enzyme activity of mycorrhizal plants without *F. solani* inoculation increased in the later stage (**Figure 6D**). *R. intraradices* inoculation significantly increased the activity of polygalacturonase, *R. intraradices* inoculation significantly increased the activity of polygalacturonase, and the enzyme activity of *R. intraradices* and pathogen inoculation treatment was higher than that of pathogen inoculation treatment (**Figure 6C**) in five periods.

## 4 Discussion

In this study, pot experiments were conducted to investigate the effects of *R. intraradices* on the growth, related physiological changes and root rot resistance of *L. barbarum*. The results showed that the colonization rate of mycorrhizal plants increased gradually with the change of treatment time, but the presence of *F. solani* showed a negative effect on the colonization of *R. intraradices*. The reason may be that there was a direct interaction between the two, or the infection of *F. solani* caused poor growth of *L. barbarum* plants and reduced the nutrient supply to *R. intraradices*, which slowed down the growth of *R. intraradices*, thus resulting in a decrease in mycorrhizal infection rate. In addition, the biomass and chlorophyll content of mycorrhizal *L. barbarum* increased at different degrees, indicating that *R. intraradices* promoted the growth of plants by facilitating the uptake of water and nutrients from the soil, which was consistent with the results with other reports showing that *R. intraradices* increasing the absorption of nutrients by host plants through mycelium, thereby promoting the growth of *Ambrosia artemisiifolia* and colonization in the roots of *Cinnamomum migao* seedlings and promoting their growth and thereby reducing the incidence and disease index of root rot, and indirectly enhance the resistance of *L. barbarum* to root rot (Liao et al., 2021; Kong et al. 2021).

Phenylalanine is one of the final products of the shikimic acid pathway. Phenylpropanoid metabolism produces more than 8000 aromatic compounds. The reaction of the PPP is catalyzed by PAL, C4H, and 4CL. The three-step catalytic reaction is considered to be the core reaction of the entire metabolic pathway and directly determines whether the PPP can proceed smoothly (Fraser et al., 2011). There are many downstream branches of the PPP, among which the lignin and flavonoid metabolic pathways are the two main branches studied in this paper. PAL is a key enzyme and rate-limiting enzyme in phenylpropanoid metabolism, which plays an important role in plant growth and development, disease resistance and stress resistance (Zhang et al., 2023b). C4H catalyzed the hydroxylation of cinnamic acid to p-coumaric acid. Then p-coumarate coenzyme A was formed under the catalysis of 4-coumarate-CoA ligase. These compounds were transformed into various phenylpropanoids as substrates for the next reaction. Our study found that the PAL, 4CL and C4H of mycorrhizal diseased plants were significantly higher than those of non-mycorrhizal diseased plants, indicating that *R. intraradices* inoculation could activate the PAL, 4CL and C4H activities in *L. barbarum* root rot plants, thus accelerating the process of PPP. This was consistent with the results of AMF inoculation increased PAL activity in pepper leaves and mechanically damaged tomato leaves (Song et al., 2018; Oliveira et al., 2022).

Other catalytic enzymes in the PPP of plants, such as HCT, are considered to be a key factor in controlling the direction of downstream metabolism of monophenolic compounds, and CHI and CHS serve as key enzymes in the downstream branching flavonoid metabolic pathway. Our study found that the activities of HCT, CHI and CHS in *L. barbarum* plants inoculated with *R. intraradices* were significantly higher than those of non-inoculated plants. Studies have found that C4H, CHI, 4CL, and PAL activities and *PtPAL1* and *Pt4CL* expressions were induced by *Funneliformis mosseae* under water stress (Liu et al., 2022), which was similar to the results of this study. At the same time, the plant PPP generally participates in disease resistance immunity through two aspects. On the one hand, synthesizing lignin promotes the degree of cell lignification and forms a physical barrier to prevent the invasion of pathogenic fungi. On the other hand, it produces a variety of metabolites such as phenols and flavonoids to inhibit the growth of pathogenic fungi (Shang et al., 2022).

The products after the above reaction are used as precursors to produce total phenols, flavonoids and lignin under the catalysis of enzymes. Our study found that *R. intraradices* inoculation increased the content of lignin, total phenols and flavonoids in *L. barbarum* within a certain treatment time range, and enhanced its disease resistance. However, the lignin content in the leaves was not significantly different in the early stage. It was speculated that it may be related to the infection of *F. solani* and the fact that the disease site of the plant is in the root, which has little effect on the above-ground part. In the later stage, the accumulation of lignin inhibited the reproduction of *F. solani*. The increase in flavonoid content is of great significance to improve the disease resistance of *L. barbarum*. Phenolic substances can further oxidize polyphenols into quinones that are highly toxic to pathogens and have a certain inhibitory effect on pathogen production (Hua et al., 2017). This was consistent with the results of *Pichia guilliermondii* promoting the accumulation of total phenols, total flavonoids and main monomeric phenolic compounds by increasing the enzyme activity and gene expression level of phenylalanine ammonia-lyase and 4-coumaric acid-CoA ligase, and inducing the resistance of peach fruit soft rot caused by *Rhizopus stolonifer* (Li et al., 2023).

Phytohormones play an important role in plant immunity and defense mechanisms. SA, as a phenolic plant hormone, initiates the expression of disease-related genes and participates in the synthesis of defensive compounds involving local resistance and systemic acquired resistance. It can regulate a variety of plant growth and development phenotypes, including seed germination, fruit ripening, senescence, and defense responses to biotic and abiotic stresses (Wani et al., 2017). This study showed that the SA level of *L. barbarum* plants increased. When infected by *F. solani*, the SA level of mycorrhizal *L. barbarum* plants was significantly higher than that of non-mycorrhizal plants, indicating that the content of endogenous hormones was significantly affected after *F. solani* infected plants. However, the presence of *R. intraradices* can increase the content of plant hormones, and is more effective in controlling soil-borne pathogens. The trend of JA content in the later stage was slightly different from that of SA, probably because as an important signal molecule in plant defense response, SA and JA-mediated pathways were relatively independent and antagonistic at some sites. However, SA and JA are not opposite, and there are still intersections in the process of regulating plant disease resistance. Ambarwati et al. (2022) found that mycorrhiza enhanced the resistance of tropical pepper to *Ralstonia solanacearum* by increasing the relative gene expression levels of JA and SA. Fujita et al. (2024) showed that mycorrhizal colonization activated the tomato immune system, resulting in higher expression levels of SA and JA-related defense genes, which were similar to our study.

In the plant-activated defense system, in addition to activating the defense enzyme protection system, it also includes the induction of pathogenesis-related proteins. Chitinase is a pathogenesis-related protein, and its hydrolysate chitin oligosaccharides can be used as an elicitor to induce defense responses in plants, thereby inhibiting the growth of pathogens (Chen et al., 2022). Pathogenic fungal infection can induce the rapid accumulation of β-1,3-glucanase in plants, which is one of the main defense responses of plants. When plants are invaded by pathogens, the secreted chitinase and β-1,3-glucanase can decompose chitin and β-1,3-glucan exposed to the tips of fungal hyphae, thus directly participating in the process of plant disease resistance (Li et al., 2004). The production of polygalacturonase and pectin methylgalacturonase can not only form complexes with pectin, an important component of the cell wall but also specifically recognize cell wall degrading enzymes of pathogens, thereby eliminating pathogens and reducing the damage of pathogens to plants (Davidsson et al., 2017). In this study, when the pathogen invaded the plant, the activities of chitinase and β-1,3-glucanase in mycorrhizal *L. barbarum* were significantly higher than those in diseased plants, which was consistent with the results of Pu et al. (2022) and Wang et al. (2021). At the same time, the activities of pectin methylgalacturonase and polygalacturonase were also significantly different. Zhang and Tang (2021) showed that inoculation with AMF could increase the activity of polygalacturonase in *P. cathayana* to inhibit the growth of mycelia, reduce the damage of pathogens to *P. cathayana*, and improve the disease resistance of *P. cathayana*, which was similar to this study.

The mechanisms by which AMF improve plant disease resistance include competing with pathogens for nutrients, regulating host endogenous hormones, and activating host defense systems (increasing host defense enzyme activity, inducing the synthesis of pathogenesis-related proteins and secondary metabolites). In this paper, through the determination of the above indicators, it is helpful to better understand the growth-promoting effect and disease resistance mechanism of *R. intraradices* as a biocontrol microorganism on *L. barbarum* plants and to evaluate the role and value of *R. intraradices* to a certain extent. ln summary, mycorrhizal colonization can enhance the ability of *L*. *barbarum* to resist root rot by inducing the activation of the phenylpropane metabolic pathway and increasing the content of pathogenesis-related proteins and plant signaling substances. It is suggested that *R. intraradices* plays a beneficial role in sustainable agriculture by symbiotically associating with *L. barbarum*. However, the internal molecular mechanism of how *R. intraradices* induces and activates the PPP in *L. barbarum* plants is still unclear. The differential gene expression of *L. barbarum* plants treated with *R. intraradices* can be further studied by transcriptomics technology, to analyze the roles of key differential genes and key pathways, find the relationship between key differential genes, and verify the mechanism of *R. intraradices* treatment to improve the resistance of *L. barbarum* plants to root rot at the molecular level.

## 5 Conclusion

*R. intraradices* could establish a stable symbiotic relationship with the roots of *L. barbarum*, promote the accumulation of biomass, increase the chlorophyll content, reduce the incidence and disease index of root rot, and enhance the activity of phenylpropanoid metabolism-related enzymes, the content of pathogenesis-related proteins and the content of plant signal substances in the leaves of *L. barbarum*. The results showed that *R. intraradices* inoculation treatment increased the activity of phenylpropanoid metabolism-related enzymes and the content of resistant substances in *L. barbarum* plants, thereby enhancing the resistance of *L. barbarum* plants to root rot.

## Data Availability

The original contributions presented in the study are included in the article/supplementary material. Further inquiries can be directed to the corresponding author.
